# Predictive value of retinal oximetry, optical coherence tomography angiography and microperimetry in patients with treatment-naïve branch retinal vein occlusion

**DOI:** 10.1186/s40942-023-00468-7

**Published:** 2023-06-28

**Authors:** Katrine Hartmund Frederiksen, Frederik Nørregaard Pedersen, Anna Stage Vergmann, Dawei Yang, Caroline Schmidt Laugesen, Jesper Pindbo Vestergaard, Torben Lykke Sørensen, Carol Y Cheung, Ryo Kawasaki, Tunde Peto, Jakob Grauslund

**Affiliations:** 1 Department of Ophthalmology, Odense University Hospital, Odense, Denmark; 2 Department of Clinical Research, University of Southern Denmark, Odense, Denmark; 3 Department of Ophthalmology and Visual Sciences, The Chinese University of Hong Kong, Hong Kong Special Administrative Region, Hong Kong, China; 4 Department of Ophthalmology, Zealand University Hospital, Roskilde, Denmark; 5 Faculty of Health and Medical Science, University of Copenhagen, Copenhagen, Denmark; 6 Department of Vision Informatics, Osaka University School of Medicine, Osaka, Japan; 7 School of Medicine, Dentistry and Biomedical Sciences, Queen’s University, Belfast, Northern Ireland UK

**Keywords:** Retinal oximetry, Optical coherence tomography angiography, Microperimetry, Branch retinal vein occlusion, Vascular endothelial growth factor inhibition

## Abstract

Vascular endothelial growth factor inhibitors have substantially improved the visual outcomes in patients with macular edema (ME) caused by branch retinal vein occlusion (BRVO), but treatment outcomes are highly variable and early prediction of expected clinical outcome would be important for individualized treatment.

As non-invasive metabolic, structural and functional retinal markers might act as early predictors of clinical outcomes, we performed a 12-month, prospective study aimed to evaluate if baseline retinal oximetry, optical coherence tomography angiography (OCT-A) or microperimetry were able to predict need of treatment, structural or functional outcome in patients with ME caused by treatment-näive BRVO.

We evaluated 41 eyes of 41 patients with a mean age of 69.6 years and 56% females. We found a strong tendency towards a higher retinal arteriolar oxygen saturation in patients without a need of additional aflibercept treatment after the loading phase (99.8% vs. 92.3%, adjusted odds ratio 0.80 (95% confidence interval 0.64-1.00), adjusted p = 0.058), but otherwise, retinal oximetry, OCT-A or microperimetry were not able to predict need of treatment, structural nor functional outcomes. (Trial registration: clinicaltrials.gov, S-20,170,084. Registered 24 August 2014, https://clinicaltrials.gov/ct2/show/NCT03651011*)*

## Introduction

While macular edema (ME) in branch retinal vein occlusion (BRVO) can be effectively treated with vascular endothelial growth factor (VEGF) inhibitors, the treatment burden varies widely between patients with 30–50% of patients responding insufficiently [1, 2] and one in two patients requiring long-term VEGF inhibitory treatment in order to control ME recurrence [3]. The ability to predict the expected prognosis would allow for a more individualized treatment.

Retinal oximetry is a metabolic marker associated with retinal ischemia, which is a key factor in BRVO [4]. While it has been identified as a predictor of disease activity in diabetic macular edema [5], conflicting preliminary results have been presented in BRVO [6, 7], with relevant clinical outcomes not sufficiently examined.

While optical coherence tomography angiography (OCT-A) might predict progression of diabetic retinopathy [8] and visual function in retinal vein occlusion (RVO) [9], its ability to predict need of treatment or structural outcome in BRVO patients has not been evaluated. Likewise, a retrospective study of retinal microperimetry indicates potential associations with visual acuity outcomes after anti-VEGF treatment [10], but there have been no prospective studies evaluating any potential predictive value on treatment need and structural outcomes.

Thus, we aimed to examine the predictive value of retinal metabolic, structural and functional measures, measured by retinal oximetry, OCT-A and microperimetry, in determining need of treatment, structural and functional 12-month outcomes in patients with treatment-naïve BRVO treated with aflibercept.

## Materials and methods

The data for this prospective observational study derived from a previously reported, prospective, 12-month, randomized controlled clinical trial, including 41 eyes of 41 patients with treatment-naïve BRVO[11]. We recruited patients from August 2018 to August 2020, based on the following criteria: centre-involved ME due to BRVO diagnosed within 6 months in patients ≥ 18 years of age, with a best corrected visual acuity (BCVA) between 35 and 80 Early Treatment Diabetic Retinopathy Study (ETDRS) letters and central retinal thickness (CRT) > 300 μm, as measured by optical coherence tomography (OCT). We excluded patients with active retinal or iris neovascularization in the study eye, clouding conditions preventing laser treatment, other potential causes of ME, prior anti-VEGF treatment or macular laser photocoagulation in the study eye or uncontrolled, untreated hypertension (blood pressure ≥ 160/110 mmHg). Likewise, patients were excluded from analysis of retinal oximetry if they had ocular or systemic conditions expected to affect oxygen saturation measurements [12] or if image quality was low.

At baseline, the diagnosis was confirmed by a full medical history and ophthalmic evaluation including BCVA measured with ETDRS charts (Precision Vision, Illinois, USA), macular OCT line scan by Spectralis (Heidelberg Engineering GmbH, Germany), slit lamp examination and 50 degrees macula-centred fundus photographs (TRC-50DX fundus camera, Topcon, Tokyo, Japan). Furthermore, patients were subjected to retinal oximetry (Oxymap T1, Oxymap, Iceland), OCT-A using a swept-source OCT (Topcon 3D OCT 2000, TRITON, Topcon, Japan) and microperimetry (MP-3, NIDEK, Japan). All patients then received three monthly intravitreal injections of 2.0 mg aflibercept, followed by navigated central laser in one group (n = 21) and no laser in the other group (n = 20). From month four through twelve, patients were followed up monthly with BCVA and OCT, and re-treated with aflibercept following an as-needed regimen (if CRT increased > 20% compared to lowest measurement or if a BCVA loss of above five letters compared to baseline was recorded).

As we detected no differences in structural or functional outcomes between the two treatment groups [11], patients were pooled for analysis in the present study.

## Retinal oximetry

We acquired images as previously described in detail elsewhere [13]. In brief, an optic-disc-centred image was captured by the Oxymap T1, and beam-splitted into two different wavelengths, one where absorbance of light is unaffected and one where absorbance of light is affected by the hemoglobin oxygenation. From these, an estimated, relative oxygen saturation of the retinal vessels was calculated. Image analysis was performed in accordance with instructions by the manufacturer [14] using the build-in software (Oxymap Analyzer 2.5.0, Oxymap, Iceland). Briefly, measurements were performed by placing two circles around the optic disc, one at 1.5 disc diameters and one at three disc diameters, within which all measurements were performed. One large arteriole and venule of each quadrant were automatically traced at a length between 50 and 200 pixels. Measurements were presented as the average saturation of the four traced arterioles and venules, respectively.

## OCT angiography

Macula-centred, 4.5 × 4.5 mm OCT-A scans were obtained by a trained physician. Individual images of the superficial capillary plexus (SCP) and the deep capillary plexus (DCP) were extracted using Imagenet I-base version 3.25.0 (Topcon Europe Medical B.V., The Netherlands). These were processed and analyzed using MATLAB (MathWorks, Natick, MA), as described [15], providing quantitative OCT-A metrics including foveal avascular zone (FAZ) area, non-perfusion area, vessel density and fractal dimension. A post-analysis image check was performed, ensuring correct detection of areas by the software, and any images with faulty delineation, were disregarded from further analysis.

## Microperimetry

The microperimetry analysis was described in details elsewhere [11]. In short, patients were subjected to a stimuli pattern of 45 points within the central 12 degrees of the macula. The stimuli were white, Goldmann III sized, with a starting threshold of 12 dB, 200 ms duration and a 4-2-1 staircase strategy. Automatic retinal focusing and alignment as well as pausation of measurements during eye movements was utilized. The threshold of the 45 points were averaged and presented as mean retinal sensitivity.

### Statistical analysis

Data were described at baseline with number and percentage for categorical variables and mean ± standard deviation or median (25th ;75th percentile) for continuous variables, as appropriate. We performed a logistic regression for each predictive biomarker on three binary outcomes; injection need after loading phase, ME (defined as CRT < 300 μm) at month 12, and change in BCVA of 10 or more letters from baseline. The model was adjusted for sex (male/female), age (continuous), BCVA at baseline (continuous) and CRT at baseline (continuous), and results were reported as mean baseline value ± SD of each biomarker (Table 1) and adjusted odds ratio (OR) with 95% confidence intervals (CI) and p-values of each biomarker (Table 2). The statistical significance level was set at 0.05, and data analysis was performed using Stata 17.0 (StataCorp, College Station, TX, USA).

All participants provided written informed consent. The study was approved by the Regional scientific ethics committee (approval S-20,170,084), registered at ClinicalTrials.gov (NCT03651011) and followed the tenets of the declaration of Helsinki.

## Results

We included 41 eyes of 41 patients. Mean age was 69.6 ± 10.0 years, 56% of patients were female, and baseline BCVA was 70.0 [62.0;75.0] ETDRS letters, while baseline CRT was 502 [449;580] µm [11].

In analysis of retinal oximetry measures, two patients were excluded due to glaucoma of the affected eye. In OCT-A, 14 SCP images and 33 DCP images were excluded due to low quality, blurry picture, signal loss, poor centration or artefacts (e.g. due to cystoid ME) resulting in mis-tracing by the software. Seven further SCP images were excluded from analysis of FAZ area, due to faulty delineation of FAZ. The number of acceptable DCP images was too low to analyze further. In microperimetry, two patients could not cooperate to fulfill the test and were excluded.

We report numerical differences in baseline values according to outcome group, primarily in arteriolar saturation and area of FAZ in all three outcomes (Table 1), none of which reach statistical significance. The most promising marker was retinal arteriolar saturation, in predicting need for re-treatment, with an OR of 0.80 (95% CI 0.64 to 1.00) (adjusted p = 0.058). Thus, a higher arteriolar retinal saturation tended to decrease the likelihood of needing additional aflibercept after loading phase.

**Table 1:** Baseline value of novel markers by outcome group in patients with BRVO treated with aflibercept

	Need of additional aflibercept after loading		Macular edema at month 12		Improvement of 10 or more ETDRS letters from baseline	
	No	Yes	No	Yes	No	Yes
*n*	10	31	26	15	*13*	28
*Retinal oximetry (n = 33)*						
Arteriolar saturation (%)	99.8 ± 9.0	92.3 ± 10.1	96.8 ± 10.8	90.0 ± 8.0	90.7 ± 9.3	95.9 ± 10.4
Venular saturation (%)	53.5 ± 14.2	52.3 ± 16.9	55.9 ± 13.7	47.6 ± 18.5	54.7 ± 12.4	51.7 ± 17.5
*OCT-A, SCP*						
Area of FAZ (mm^2^) *(n = 20)*	0.28 ± 0.12	0.31 ± 0.16	0.36 ± 0.17	0.24 ± 0.08	0.28 ± 0.11	0.32 ± 0.17
Area of non-perfusion (mm^2^) *(n = 27)*	4.43 ± 1.07	4.48 ± 1.36	4.53 ± 1.06	4.35 ± 1.69	4.55 ± 1.42	4.43 ± 1.23
Vessel density (%) *(n = 27)*	73.8 ± 6.7	73.2 ± 8.7	73.2 ± 6.5	73.7 ± 11.1	72.8 ± 9.7	73.7 ± 7.4
Fractal dimension *(n = 27)*	1.68 ± 0.01	1.68 ± 0.02	1.68 ± 0.01	1.68 ± 0.02	1.68 ± 0.02	1.68 ± 0.01
*Microperimetry (n = 39)*						
Mean retinal sensitivity (dB)	22.6 ± 3.4	20.9 ± 4.2	21.3 ± 4.1	21.3 ± 4.1	22.5 ± 4.4	20.8 ± 3.9

Presented as mean ± SD. *BRVO = branch retinal vein occlusion, SCP = superficial capillary plexus, FAZ = foveal avascular zone, dB = decibel, ETDRS = early treatment diabetic retinopathy study.*

However, no statistically significant effect of any of the analyzed biomarkers, in predicting either re-treatment need, edema at months 12 or improving 10 or more letters in BCVA from baseline was found (Table 2).

**Table 2:** Logistic regression results of novel markers, in predicting 12 month outcomes in patients with BRVO treated with aflibercept

		Need of additional aflibercept after loading		Macular edema at month 12		Improvement of 10 or more ETDRS letters from baseline	
	Predictor increment	Adjusted odds ratio (95% CI)	Adjusted p-value	Adjusted odds ratio (95% CI)	Adjusted p-value	Adjusted odds ratio (95% CI)	Adjusted p-value
*Retinal oximetry (n = 33)*							
Arteriolar saturation (%)	1%	0.80 (0.64;1.00)	0.054	0.94 (0.85;1.03)	0.175	1.05 (0.96;1.16)	0.265
Venular saturation (%)	1%	0.96 (0.89;1.04)	0.379	0.97 (0.91;1.03)	0.282	1.00 (0.94;1.06)	0.938
*OCT-A, SCP*							
Area of FAZ (mm^2^) *(n = 20)*	0.01 mm^2^	0.79 (0.55;1.12)	0.187	0.88 (0.76;1.03)	0.105	1.08 (0.98;1.19)	0.125
Area of non-perfusion (mm^2^) *(n = 27)*	0.01 mm^2^	0.62 (0.20;1.92)	0.409	0.86 (0.34;2.15)	0.748	0.76 (0.30;1.90)	0.554
Vessel density (%) *(n = 27)*	1%	1.08 (0.89;1.30)	0.428	1.01 (0.87;1.18)	0.865	1.06 (0.91;1.23)	0.481
Fractal dimension *(n = 27)*	0.01	1.02 (0.32;3.22)	0.973	0.89 (0.37;2.13)	0.799	2.02 (0.82;4.98)	0.124
*Microperimetry (n = 39)*							
Mean retinal sensitivity (dB)	1 dB	1.00 (0.70;1.44)	0.982	1.08 (0.79;1.46)	0.631	0.96 (0.68;1.35)	0.800

Presented as odds ratio with 95% confidence interval and p-value of logistic regression model adjusted for age, sex, baseline central retinal thickness and baseline best corrected visual acuity of the variable in question. Predictor increment are the increment of the given variable for which the odds ratio apply.

*BRVO = branch retinal vein occlusion, SCP = superficial capillary plexus, FAZ = foveal avascular zone, dB = decibel, ETDRS = early treatment diabetic retinopathy study.*

## Discussion/Conclusion

In this prospective study of patients with BRVO, we found a tendency of retinal arterial saturation to predict need for re-treatment in a multivariable model including established biomarkers, with a higher baseline saturation in patients with no need for additional aflibercept after loading phase (99.8 ± 9.0% vs. 92.3 ± 10.1%, OR 0.80 (95% CI 0.64 to 1.00), adjusted p = 0.054). This, however, was not statistically significant. We report no significant effect of retinal arteriolar or venular saturation, OCT-A measures or mean retinal sensitivity in predicting re-treatment need, structural or functional outcomes after 12 months of treatment with aflibercept.

Retinal oximetry have been proven a useful biomarker in eyes with central retinal vein occlusion (CRVO), with reports of lower venous saturation in affected eyes [16–18], and increases in venous saturation following anti-VEGF treatment [17, 18]. In BRVO, increased arteriolar saturation of affected vessels have been consistently reported [16, 19, 20]. A recent study found no predictive value of venous oxygen saturation on visual acuity outcomes in BRVO after treatment [7]. They did not evaluate predictive value of arteriolar saturation, whereas we report a tendency of arteriolar saturation to predict need for re-treatment with aflibercept. This finding seems paradoxical to recent findings of lower linear blood flow velocity being associated with higher retinal saturation measurement [21], and with another study finding correlation between larger area of affected retina and higher arterial saturation [7], thus the mechanism behind our results remain unclear.

OCT-A is a promising biomarker in other retinal diseases, including diabetic retinopathy [8], but only few studies evaluated predictive value of OCT-A parameters at baseline in BRVO with ME. Winegarner et al. [9] conducted a retrospective study and found associations between area of FAZ and mean vessel density with visual outcome, proving feasibility in another imaging system. Contrary to this, we report no association with functional outcome, nor on re-treatment need and functional outcomes. We found that the applicability of OCT-A in BRVO with ME is limited at baseline by trouble obtaining and analyzing at sufficient quality, and as a quantitative modality it might be more useful after resolution of ME.

To the best of our knowledge retinal sensitivity by microperimetry has not previously been evaluated as a marker of re-treatment need or structural outcome in retinal vein occlusion, but a previous study report retinal sensitivity as useful in predicting BCVA after three months [10]. Contrary to this, we report almost no value as an added biomarker in adjusted analysis of increase in BCVA of 10 or more letters, which might be attributed to the difference in definition of visual acuity outcome and longer follow-up time.

Although our study was strengthened by its prospective design with 12 months of follow-up, limitations include a limited sample size and difficulties obtaining high-quality baseline images given disease-induced presence of intraretinal hemorrhages and ME. Especially in OCT-A, a certain amount of images had to be excluded, which might bias results.

In conclusion, we report a strong tendency between higher retinal arteriolar saturation and no need of subsequent treatment in patients with treatment-näive BRVO and encourage that this is verified in long-term studies of larger cohorts. In contrast, retinal vascular structure or function evaluated by OCT-A and microperimetry did not associate with treatment burden, functional nor structural outcome and is not likely to act as early decision-support for planning of clinical care.

## Data Availability

The data that support the findings of this study are not publicly available due to general data protection regulation.

## List of Abbreviations

BCVA: Best corrected visual acuity
BRVO: Branch retinal vein occlusion
CRT: Central retinal thickness
ETDRS: Early treatment diabetic retinopathy study
FAZ: Foveal avascular zone
ME: Macular edema
OCT: Optical coherence tomography
OCT-A: Optical coherence tomography angiography
RVO: Retinal vein occlusion
VEGF: Vascular endothelial growth factor inhibitor
